# Supplementary material for: The Development of the Head Direction System before Eye Opening in the Rat
Source: Curr Biol. 2015 Feb 16;25(4):479–83. doi: 10.1016/j.cub.2014.12.030 (PMC4331281; doi:10.1016/j.cub.2014.12.030)
Supplement: Document S1. Supplemental Experimental Procedures and Figures S1 and S2 [file mmc1.pdf]

Current Biology
Supplemental Information

The Development of the Head Direction System before Eye Opening in the Rat

Hui Min Tan, Joshua Pope Bassett, John O'Keefe, Francesca Cacucci, and Thomas Joseph Wills

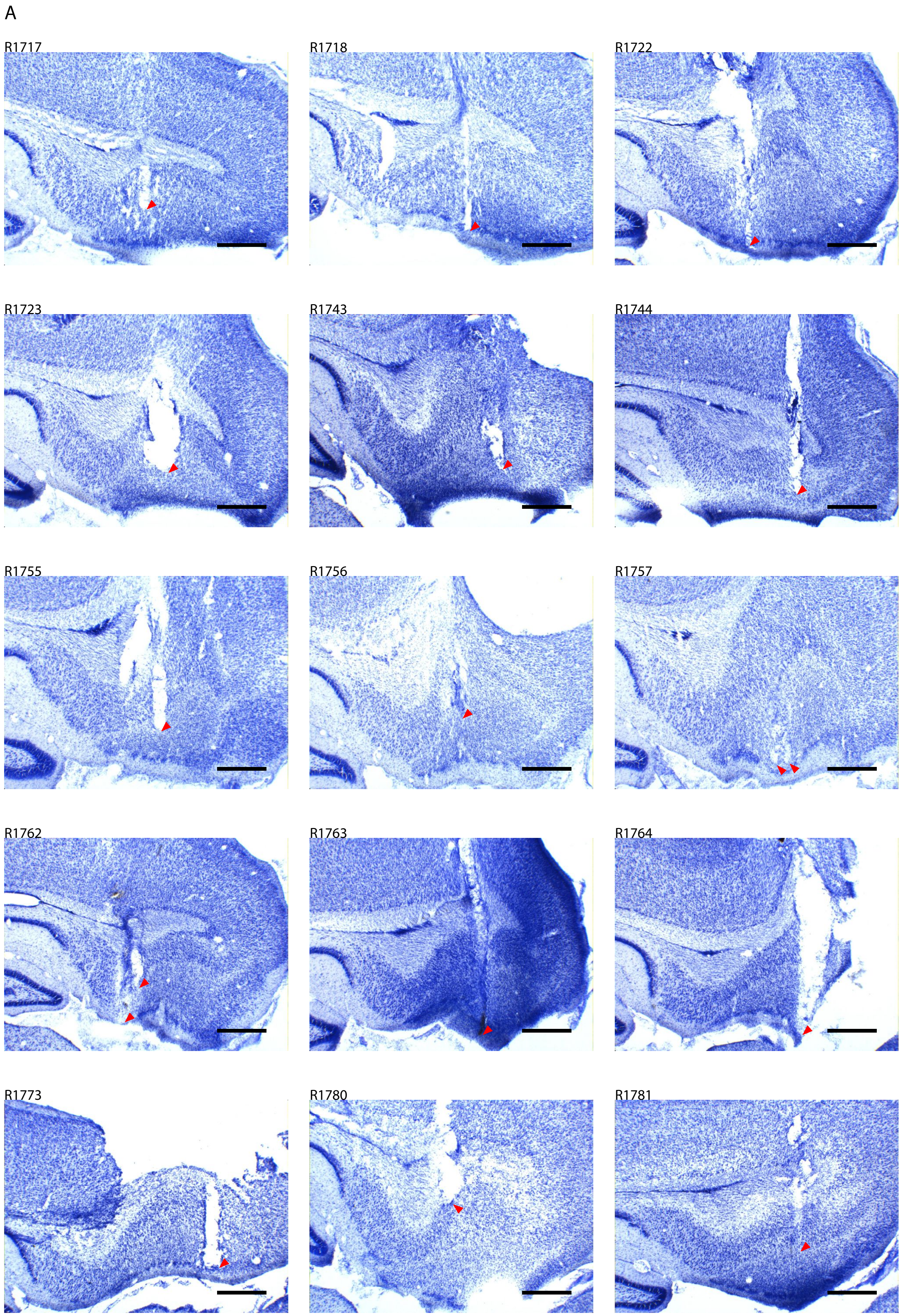
A
R1717
R1718
R1722
R1723
R1743
R1744
R1755
R1756
R1757
R1762
R1763
R1764
R1773
R1780
R1781

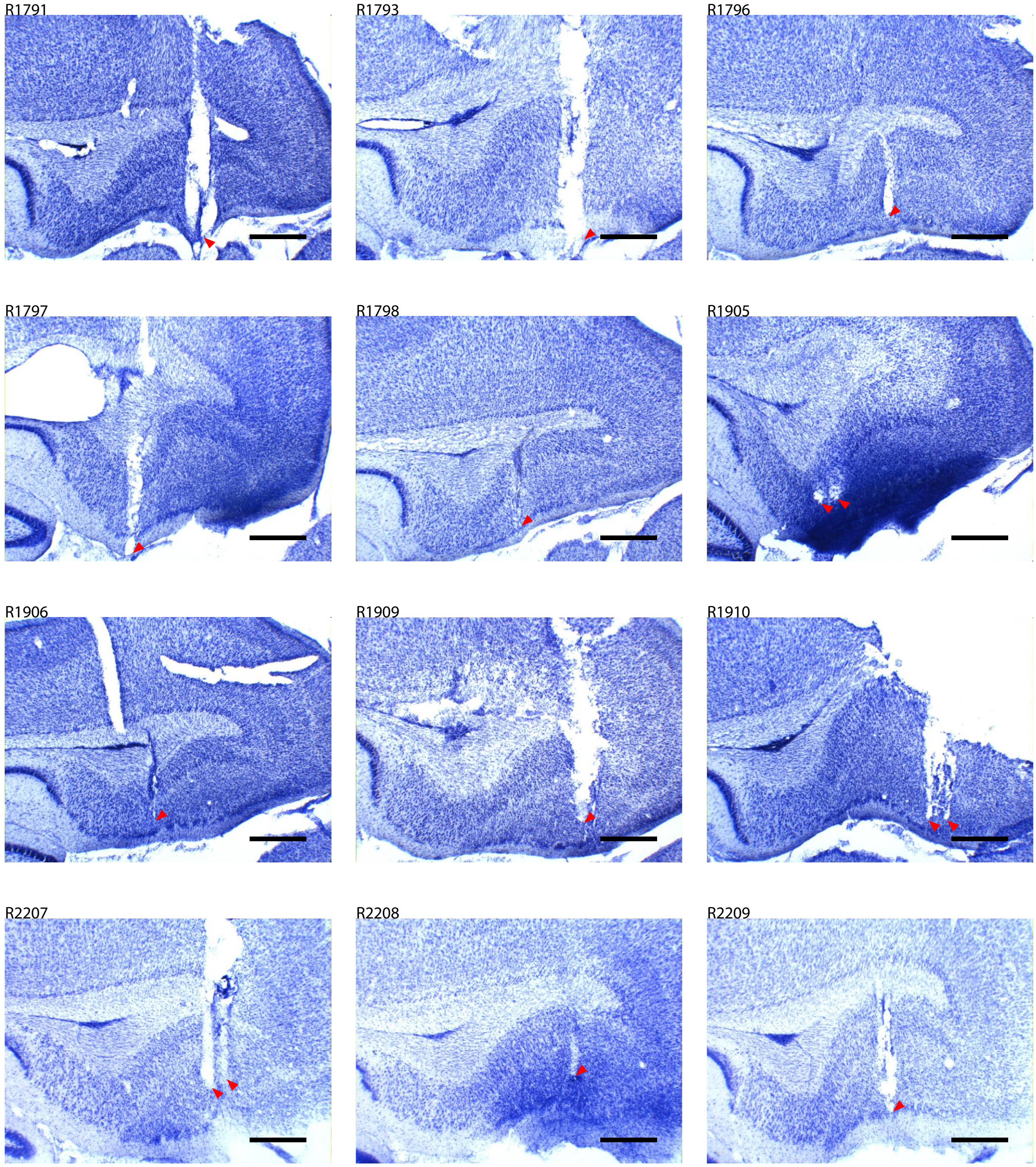
R1791
R1793
R1796
R1797
R1798
R1905
R1906
R1909
R1910
R2207
R2208
R2209

B

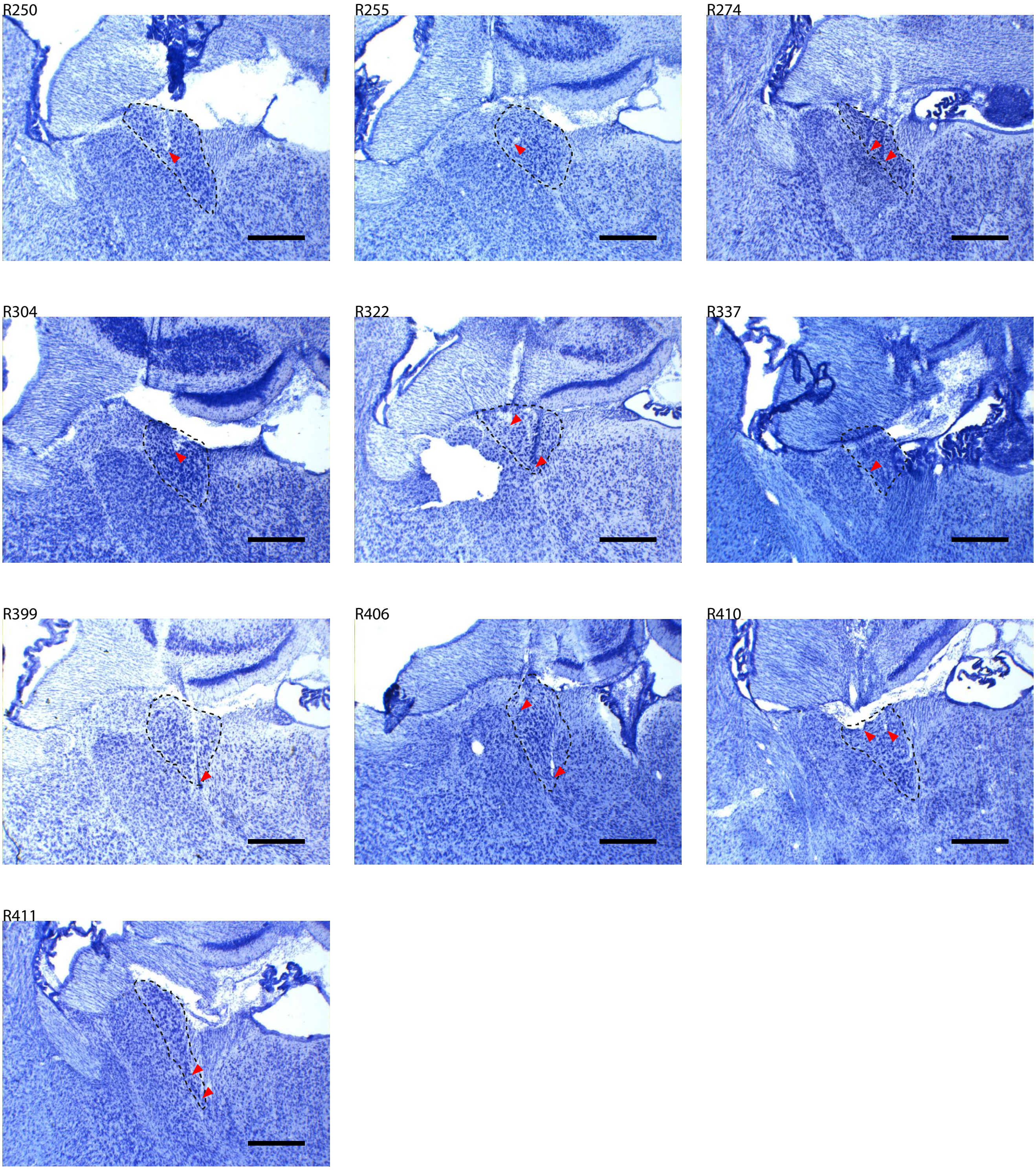

Figure S1, related to Figure 1. Nissl-stained brain sections showing representative recording locations in the PrSd (sagittal sections, n=27, panel A) and ADN (coronal sections, n=10, panel B). Red arrows mark the deepest location through which any given tetrode passed within the PrSd or ADN (the limits of the ADN are traced by dashed lines). Scale bars represent 500um.

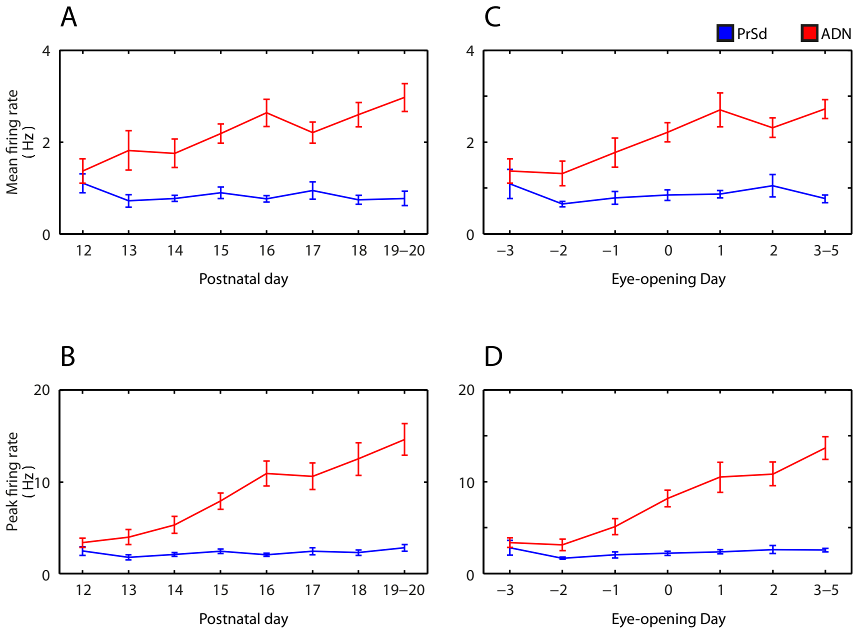

Figure S2, related to Figure 2. Firing rates of ADN HD cells increase during development to higher levels than those of PrSd HD cells. (A,C) Mean firing rates (mean ±SEM) of HD cells recorded from PrSd (blue) and ADN (red), grouped by postnatal day (Panel A) or eye-opening day (Panel C). (B,D) Peak firing rates (mean ±SEM) of HD cell polar plots from PrSd and ADN, grouped by postnatal day (Panel B) or eye-opening day (Panel D).

Supplemental Experimental Procedures

Subjects 37 male Lister Hooded rats (PrS n = 27; ADN n = 10), aged P10-P20, weighing 18-29g at the time of surgery, were used as subjects. Litters were bred on-site and implanted subjects remained with their mothers and litter-mates throughout the experimental period. Litters were housed in 42x32x21cm cages furnished with nesting material and environmental enrichment objects, and maintained on a 12:12 hour light:dark schedule with lights off at 13:00. Litters were culled to 8 pups at P4 in order to minimise inter-litter variability. Implanted pups were separated from the dam and littermates for around 20 to 120 minutes per day for electrophysiological recordings. Pups were checked at the beginning and end of each day for evidence of eye-opening. The first day on which at least one of the eyelids had opened was labelled E0.

Surgery and electrodes Rats were anaesthetised using 1-3% isoflurane and buprenorphine via subcutaneous injection at 0.15mg/kg of body weight. Rats were implanted with 4-8 tetrodes consisting of HM-L coated 90% platinum/10% iridium 17µm wire (California Fine Wire, Grover City, CA). The implanted apparatus weighed 1 gram. Tetrode bundles were implanted at the following stereotaxic coordinates, for ADN: 1.7 mm posterior to bregma, 1.2mm lateral from the midline at bregma, and 4.2mm ventral from bregma. For PrSd: 1.6mm anterior to the sinus; 2.45 mm lateral from the midline at the sinus and 2.15mm ventral to the cortical surface. Following surgery, rats were placed on a heating pad until they could move spontaneously and were then returned to the home cage. After experiments were completed, tetrode positions were confirmed by transcardially perfusing the rat (4% Paraformaldehyde in PBS) whilst the tetrodes remained in their final position, followed by brain sectioning at 30um, and Nissl-staining of the resulting sections.

Single unit recording Following surgery, rats were allowed 24 hours recovery. Tetrode bundles were then advanced ventrally in increments of 62.5-250 µm/day. Experimental recording sessions began when any single unit neural activity could be identified. Single unit data were acquired using the DACQ system (Axona Ltd, St. Albans, UK). Position and directional heading were recorded using a 2-point tracking system consisting of 2 LEDs spaced 7 cm apart and attached to the headstage amplifier in a fixed orientation relative to the animals' head. Isolation of single units from tetrode-recorded data was performed manually on the basis of peak-to-trough amplitude, or principal components using the TINT software package (Axona Ltd., St Albans, UK) with the aid of KlustaKwik [1] automated clustering. When two or more recording sessions were performed in the same day, isolated single units were treated as distinct cells if a) electrodes had been moved at least 125µm between sessions, b) at least 2 hours had elapsed between sessions, and c) the distributions of spike clusters in

waveform amplitude plots were clearly unrelated between the two sessions. Overnight, even if electrodes had not been moved, distributions of spike clusters were rarely related between the two days. Single units recorded across days were treated as independent cells unless there was similarity in either a) distributions of spike clusters on a tetrode or b) the spatial firing correlates of the cells. If single units were found to be repeatedly sampled over sessions, only data from the first session were included in the analyses. However, due to the inherent limitations of the tetrode recording technique, it should be noted that a small number of the single units in this study may represent re-sampled neurons, notwithstanding the above precautions.

Behavioural Testing Single-unit recording trials took place in one of two recording arenas. (1) To test for the presence of HD cells, and assess their spatial firing properties, recordings were performed in a square box (62.5cm side length, 50cm high) painted light grey, placed on a black platform. The box was placed in the open laboratory, and distal visual cues were available in the form of the fittings and contents of the laboratory. The floor of the arena was not cleaned. There were no further polarising cues placed within the recording arena. Rats were subject to between 1 and 4 standard recording trials per session. (2) To assess whether HD cells followed a visual landmark rotation, recordings were performed in a light-grey wooden cylinder (79cm diameter, 50cm high) placed on a black platform, centred within a set of black curtains hanging from a circular track on the ceiling. A single, spatially-polarising cue in the form of a white card measuring 102 x 77 cm was hung within the curtains, but 55 cm distal to and visible above the walls of the recording arena, with a 40 watt lamp directed at the card. Every time rat pups entered the curtained enclosure, they were purposely disoriented by gently turning them in a closed opaque box and released in the cylindrical recording box always facing in the same direction with respect to the laboratory frame of reference. Rat pups were kept in a separate holding box (40 x 40 x 5cm) furnished with bedding and a heating pad in between recording trials. For landmark rotation trials, the white cue card and lamp were moved to the opposite side of the recording arena, whilst the rat remained outside of the curtains. The floor and walls of the recording arena were not rotated when the landmark was rotated, though the floor was cleaned.

Construction of polar tuning curves To minimise artefactual correlates due to under-sampling of position, data were included in further analyses only if the linear path length for the session exceeded 15.7m, and the angular path length exceeded the equivalent of 43 full head turns (values derived from the 10th percentile of the whole dataset). Directional data were sorted into 6° bins in the yaw plane. Following this, total dwell time, d, and spike count, s, for the whole trial was calculated for each directional bin. The binned dwell time and spike counts were then smoothed using a 30° boxcar filter, and the rate for each directional bin is defined as s/d.

Classification of single-units as HD cells To minimise artefactual correlates due to under-sampling, only cells which fired at least 100 spikes in a recording session were included in further analyses. The mean resultant vector length (Rayleigh vector; RV) was calculated for the polar plot of each cell as follows: The firing rates in each bin of the polar plot were treated as grouped directional data ([2], p613). The rectangular co-ordinates X and Y of the mean (resultant) vector of the polar plot were calculated as:

$$X = \frac{\sum f_i cos\ a_i}{n}$$

$$Y = \frac{\sum f_i sin\ a_i}{n}$$

where f_i is the firing rate in each polar plot bin, a_i the angle of that bin and n the total summed rate for the polar plot. The length of the resultant vector, R, was then defined as:

$$R = \sqrt{X^2 + Y^2}$$

R is limited between 0 (no angular bias) and 1 (all firing concentrated in one directional bin), and is not affected by the overall firing rate of the cell. Single units were classified as HD cells if the RV of the polar tuning curve exceeded a threshold defined as the 95th percentile of a population of RV scores derived from age- and brain area-matched spatially shuffled data [3]. Briefly, shuffled data were generated by shifting spike trains relative to position by a random amount between 20 seconds and trial duration minus 20 seconds, leaving the temporal structure of the spike train and the positional data otherwise unchanged. The shuffled data were then used to construct polar plots, as described above. This process was repeated a sufficient number of times for there to be 100,000 shuffled RV values for every 1-day age or eye-opening group, for each brain area. Single units with an RV ≥ 95th percentile of this shuffled population were defined as HD cells.

Quantitative analysis of directional signalling *Directional information* is a measure of the extent to which a cell's firing can be used to predict the direction of the animal's head in bits/spike. The estimate of the mutual information I(R|X) between firing rate R and direction X is:

$$I(R|X) \approx \sum_i p(\vec{x_i}) f(\vec{x_i}) \log_2 \left(\frac{f(\vec{x_i})}{F} \right)$$

where $p(\vec{x_i})$ is the probability for the animal facing direction $\vec{x_i}$, $f(\vec{x_i})$ is the firing rate observed at $\vec{x_i}$, and F is the overall firing rate of the cell. I(R|X) is then divided by the overall mean firing rate of the cell in the trial,

giving an estimate in bits/spike [4]. *Across-trial stability* was defined as the correlation (Pearson's r) between spatially corresponding bins from two consecutive trials, using only those bins in which firing rate >0 Hz in at least one trial. Trial pairs were used for assessing across-trial stability if a single unit was classified as a HD cell on the first trial of the pair. *Intra-trial stability* was defined as the correlation between spatially corresponding bins from the first and second half of a single trial, using only those bins in which firing rate > 0Hz in at least one half of the trial. To define the rotation of HD cell preferred direction (following visual landmark rotation, Figure 3) the polar plot for the rotated landmark condition was correlated against that for the baseline condition, whilst rotating the baseline polar plot in 6° steps. The rotation of the baseline polar plot that produced the highest r-value was taken to define the rotation of the preferred direction of the cell. Single units were used for the preferred direction rotation analysis if they were defined as HD cells in (a) the 'baseline' visual landmark condition AND (b) either or both of two 'standard' trials (outside the curtained environment) that preceded the 'baseline' visual landmark trial.

Calculation of p = 0.05 levels for the percentage of HD cells, and analysis of directional signalling P=0.05 levels for the percentage of single units classified as HD cells (dashed lines on Figure 2A, E) were generated as follows: if M polar plots per rat were analysed for a given age and brain area, then the mean percentage of HD cells expected to be found at most once per 20 experiments under the null hypothesis (non-directional firing) was defined as the 95th percentile of a binomial distribution based on M samples and a 5% success probability. The P=0.05 levels for the measures of directional signalling for each age and brain area (dashed lines, Figure 2B, C, D, F, G, H) were derived as follows: if M polar plots contributed to the mean value for a given age and area, M polar plots were sub-sampled at random from the shuffled population (see above) for that age/region and the mean value of directional signalling found. This was repeated 200,000 times, generating a distribution of the mean scores expected from populations (of size M) of randomised data. The P = 0.05 level of spatial firing was defined as the 95th percentile of this population.

Statistical Analysis Developmental trends in the spatial characteristics of HD cell firing (Figure 2B, C, D, F, G, H) were initially analysed using a 2-way ANOVA (Postnatal age, Area) or (Eye-opening day, Area) for each measure of spatial tuning or stability. Post-hoc tests were conducted using Simple Main Effects. The developmental trend for HD cell ensembles to follow a landmark rotation were assessed using the Watson-Williams test (a circular analog of ANOVA). To test whether HD cell ensembles significantly followed landmark rotation on individual days, we used the V-test [2], a variation of the Rayleigh test for non-uniformity, in which a hypothesised response direction is specified, in this case 180°. To test for changes in inter-animal

variance of mean PFD rotations between days, we used the Watson's two-sample test for homogeneity, which tests whether two circular datasets have been drawn from populations that share the same distribution [5].

Supplemental References

1. Harris, K. D., Henze, D. A., Csicsvari, J., Hirase, H., and Buzsaki, G. (2000). Accuracy of tetrode spike separation as determined by simultaneous intracellular and extracellular measurements. J.Neurophysiol. *84*, 401–414.

2. Zar, J. H. (2010). Biostatistical Analysis 5th ed. (Prentice Hall).

3. Wills, T. J., Cacucci, F., Burgess, N., and O'Keefe, J. (2010). Development of the hippocampal cognitive map in preweanling rats. Science (80-.). *328*, 1573–1576.

4. Skaggs, W. E., McNaughton, B. L., Gothard, K. M., and Markus, E. J. (1993). An information-theoretic approach to deciphering the hippocampal code. Adv Neural Inf Process Syst *5*, 1030–1037.

5. Jammalamadaka, S. R., and Sengupta, A. (2001). Topics in Circular Statistics (World Scientific).
