# Supplementary material for: The Development of the Head Direction System before Eye Opening in the Rat
Source: Curr Biol. 2015 Feb 16;25(4):479–83. doi: 10.1016/j.cub.2014.12.030 (PMC4331281; doi:10.1016/j.cub.2014.12.030)
Supplement: Document S2. Article plus Supplemental Information [file mmc2.pdf]

Report

Current Biology

The Development of the Head Direction System before Eye Opening in the Rat

Highlights

- Head direction (HD) cells exist at least 3 days before eye opening in rats
- HD signals improve sharply at eye opening and mature rapidly thereafter
- Visual inputs can exert control over the HD system soon after eye opening

Authors

Hui Min Tan, Joshua Pope Bassett, ..., Francesca Cacucci, Thomas Joseph Wills

Correspondence

f.cacucci@ucl.ac.uk (F.C.), t.wills@ucl.ac.uk (T.J.W.)

In Brief

Tan et al. reveal the existence of head direction (HD) cells several days before eye opening in the rat pup, suggesting that the HD cell network can be organized independently of patterned vision. The stability and directional tuning of HD cells improve sharply at eye opening, demonstrating the importance of vision for accurate navigation.

CrossMark

Tan et al., 2015, Current Biology *25*, 479–483
February 16, 2015 ©2015 The Authors
http://dx.doi.org/10.1016/j.cub.2014.12.030

CellPress

Current Biology 25, 479–483, February 16, 2015 ©2015 The Authors http://dx.doi.org/10.1016/j.cub.2014.12.030

Report

The Development of the Head Direction System before Eye Opening in the Rat

Hui Min Tan,[1,4] Joshua Pope Bassett,[2,4] John O'Keefe,[3] Francesca Cacucci,[2,5,*] and Thomas Joseph Wills[1,5,*]
[1]Cell and Developmental Biology, University College London, London WC1E 6BT, UK
[2]Neuroscience, Physiology and Pharmacology, University College London, London WC1E 6BT, UK
[3]Sainsbury Wellcome Centre for Neural Circuits and Behaviour, University College London, London WC1E 6BT, UK

Summary

Head direction (HD) cells are neurons found in the hippocampal formation and connected areas that fire as a function of an animal's directional orientation relative to its environment [1, 2]. They integrate self-motion and environmental sensory information to update directional heading [3]. Visual landmarks, in particular, exert strong control over the preferred direction of HD cell firing [4]. The HD signal has previously been shown to appear adult-like as early as postnatal day 16 (P16) in the rat pup, just after eye opening and coinciding with the first spontaneous exploration of its environment [5, 6]. In order to determine whether the HD circuit can begin its organization prior to the onset of patterned vision, we recorded from the anterodorsal thalamic nucleus (ADN) and its postsynaptic target in the hippocampal formation, the dorsal pre-subiculum (PrSd), before and after eye opening in pre-weanling rats. We find that HD cells can be recorded at the earliest age sampled (P12), several days before eye opening. However, this early HD signal displays low directional information content and lacks stability both within and across trials. Following eye opening, the HD system matures rapidly, as more cells exhibit directional firing, and the quality and reliability of the directional signal improves dramatically. Cue-rotation experiments show that a prominent visual landmark is able to control HD responses within 24 hr of eye opening. Together, the results suggest that the directional network can be organized independently of visual spatial information while demonstrating the importance of patterned vision for accurate and reliable orientation in space.

Results

The HD Cell Circuit Is Present at P12

We recorded 1,483 neurons from the dorsal pre-subiculum (PrSd) and 691 neurons from the anterodorsal thalamic nucleus (ADN) in rat pups aged postnatal day 12–20 (P12–P20). In total, 485 PrSd neurons and 371 ADN neurons were classified as head direction (HD) cells (33% and 54% of PrSd and ADN single units, respectively; see Experimental Procedures for classification criteria; recording locations are shown in Figure S1). Figure 1 shows polar plots of representative HD cells recorded at each sampled age from both PrSd and ADN. We recorded directional responses in rat pups as young as 12 days old, when rats still have fused eyelids and present very limited mobility [7, 8]. The number of HD cells and the quality of their directional signaling improved with age throughout the period sampled (Figures 2A–2D). An increase in HD cell firing rate was observed in the ADN, but not the PrSd (Figure S2).

Directional information (DI), a measure of the precision with which HD cells signal direction, was relatively low in younger pups, as reflected by broader directional tuning curves (Figure 1), and increased with age (Figure 2B; ANOVA age, $F_{7,837}$ = 21.75, $p < 0.001$). Similarly, both within-trial and across-trial stability were low in the youngest animals and increased with age (across trial: Figure 2C, ANOVA age $F_{7,539}$ = 21.70, $p < 0.001$; within trial: Figure 2D, ANOVA age, $F_{7,835}$ = 58.30, $p < 0.001$). The across-trial stability of early HD cells (ADN and PrSd at P12 and PrSd at P13) was less than that expected by chance (Figure 2C; see Experimental Procedures). However, the within-trial stability was greater than expected by chance at all ages (Figure 2D), demonstrating that, even at P12, HD cells can maintain a stable directional fix throughout a recording session.

HD Signaling Is Present before and Dramatically Improves at Eye Opening

Rats are born functionally blind; their eyelids open during the second week of postnatal life. In the present experimental cohort, the median eye-opening age was P15 (range: P12–P16). The developmental changes in the HD cell circuit in relation to eye opening are shown in Figures 2E–2H, where the data are grouped by eye-opening day (E) rather than by chronological age. A significant number of HD cells can be recorded from both PrSd and ADN as early as 3 days before eye opening (E-3; Figures 1 and 2E). The transition between E-1 and E0 coincided with a sharp rise in the proportion of HD cells in both brain areas, with an increase by over 50% in PrSd (E-1: 17%; E0: 28%) and over 100% in ADN (E-1: 22%; E0: 48%).

Eye opening also marks a dramatic increase in the stability and DI content of HD cells. In both brain areas, across- and within-trial stability were low and did not change significantly before eye opening (simple main effects [SMEs], E-3 versus E-1: across-trial PrSd, $p = 0.35$; across-trial ADN, $p = 0.21$; within-trial PrSd, $p = 0.56$; within-trial ADN, $p = 0.08$; Figures 2G and 2H), whereas a rapid increase in stability took place between E-1 and E0 (SMEs, E-1 versus E0: across-trial PrSd, $p < 0.001$; across-trial ADN, $p = 0.006$; within-trial PrSd, $p < 0.001$; within-trial ADN, $p < 0.001$). The DI of ADN HD cells showed the same developmental pattern, with the first significant day-on-day increase apparent between E-1 and E0 (SMEs, E-3 versus E-1: $p = 0.22$; SMEs, E-1 versus E0: $p = 0.003$; Figure 2F). For PrSd HD cells, however, the first significant improvement on pre-eye-opening DI values did not occur until E1 (SMEs, E-1 versus E1: $p = 0.006$), indicating a slower development of spatial tuning in this area.

[4]Co-first author
[5]Co-senior author
*Correspondence: f.cacucci@ucl.ac.uk (F.C.), t.wills@ucl.ac.uk (T.J.W.)
This is an open access article under the CC BY license (http://creativecommons.org/licenses/by/3.0/).

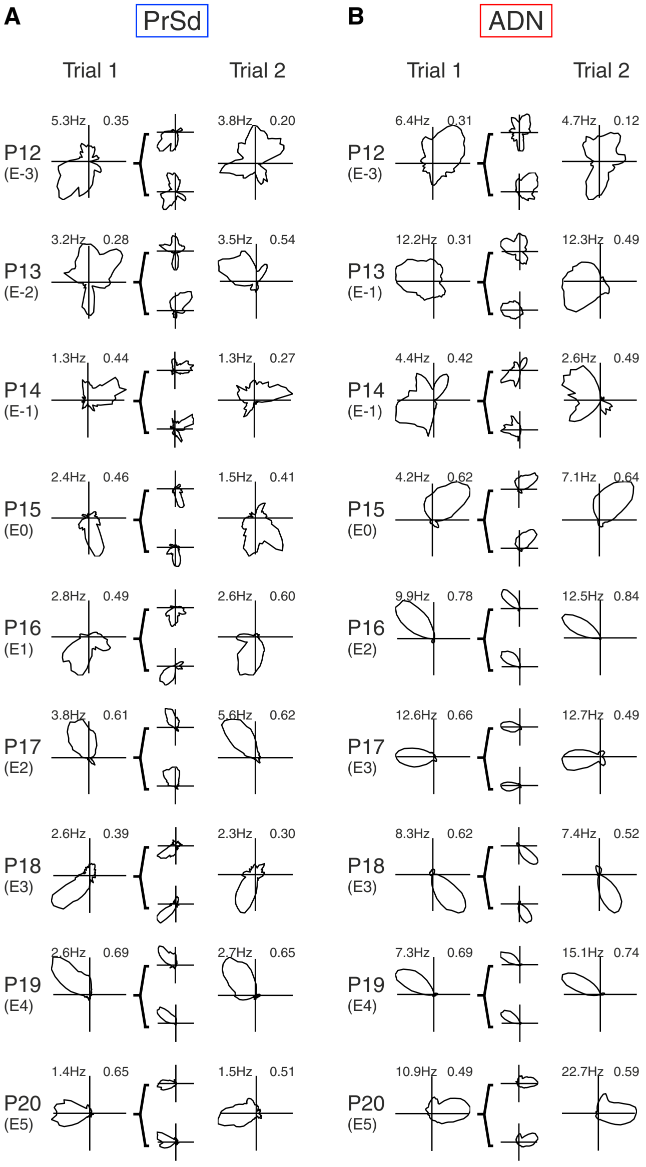

Figure 1. Representative Examples of HD Cells Recorded from PrSd and ADN

(A and B) Polar plots for two successive trials are shown for each postnatal (P) day. The eye-opening (E) day for each pair of polar plots is shown in parentheses below the postnatal day. Numbers at top left of polar plots refer to peak firing rate (Hz), and those at top right refer to Rayleigh vector length (RV; see Experimental Procedures). Directional tuning curves for the first and second halves of Trial 1 are shown within brackets as miniature polar plots. Criteria for selected cells are as follows: RV and within-trial stability for Trial 1 and across-trial stability for Trial 1 versus Trial 2 all fall within 1 SD of the population mean for the relevant age and eye-opening group. See Figure S1 for recording locations.

HD Cells Are Controlled by a Visual Landmark 1 Day after Eye Opening

In adult rats, the preferred firing direction (PFD) of HD cells is strongly influenced by visual landmarks: angular displacement of a prominent distal visual cue will commonly lead to a corresponding rotation of HD cell PFDs [4]. We tested the salience of visual input in developing animals by rotating a single prominent landmark, exclusively accessible through vision (Figure 3A; Experimental Procedures). The earliest examples of simultaneously recorded HD cell ensembles following the landmark rotation occurred at P15, corresponding to either E0 or E1 (Figures 3B and 3C). The visual landmark exerted control only on a subset of HD ensembles at E0 (Figure 3E; V = 2.29, p = 0.81; for an explanation of the V test, see Supplemental Experimental Procedures), while from E1 onward, HD cell ensembles were significantly under visual cue control (V = 4.57, p = 0.02). The variance between animals' mean PFD responses continued to decrease until E3–E5 (circular variance: E1, 0.52; E2, 0.15; E3–E5, 0.04), but there was no significant difference between the E1 and E3–E5 distributions, as assessed by Watson's two-sample test of homogeneity (n = 20, test statistic = 0.104, p > 0.1). The integration of visual information into the HD circuit therefore appears to take place soon after eye opening, with around 24 hr required for visual cues to establish control over HD cell responses.

Discussion

A Functional HD Circuit Can Be Organized Independently of Patterned Visual Input

The HD signal is foundational to an animal's representation of space, as HD cells are, together with entorhinal border cells [9], among the first spatially modulated neurons within the hippocampal mapping system to reach maturity in the rat. Previous developmental studies indicated that the HD circuit is adult-like soon after eye opening [5, 6], in the third postnatal week (~P16). The formation of the HD circuit must therefore occur during a period when the animal's ability to sample spatial features of their environment is greatly restricted by limited mobility and rudimentary sensory input [7, 8, 10].

In the adult rat, HD cells rely on information about self-generated motion, notably from the vestibular system [11, 12], to update directional heading from moment to moment in a process called angular path integration. At the same time, they incorporate information about stable landmark cues in the environment to take directional "fixes" and correct for accrued path integration error [4, 13]. Visual landmarks exert particularly strong control over HD responses in the adult rat [4, 13, 14], to the point that they often override path integrative information when a conflict between these two inputs occurs [15–17]. More recent evidence [18] indicates that optic flow information can also exert control over anterior-thalamic HD signals, reinforcing the view that vision is the dominant input in controlling HD responses.

In light of this experimental evidence, it is notable that the results presented here indicate that significant numbers of HD cells can be recorded from both PrSd and ADN in rat pups as early as 3 days before eye opening (E-3; Figure 2E; consistent with previous preliminary reports [R.F. Langston et al., 2010, FENS, abstract]; [H.M. Tan et al., 2010, Soc. Neurosci., abstract]). At this age, the nascent HD circuit is therefore likely encoding an orientation signal in the absence of visual input in the form of either discrete visual landmarks or visual velocity information from optic flow.

From at least P12 onward, HD cells are able to maintain a consistent preferred direction within a recording session. Our results suggest that during early development, HD responses can be anchored to an external reference frame using non-visual environmental cues, most likely local olfactory and tactile cues. The only study testing auditory influence on adult HD cells failed to demonstrate auditory cue control over HD cell PFDs [13], and in pups, the ear canal is sealed until

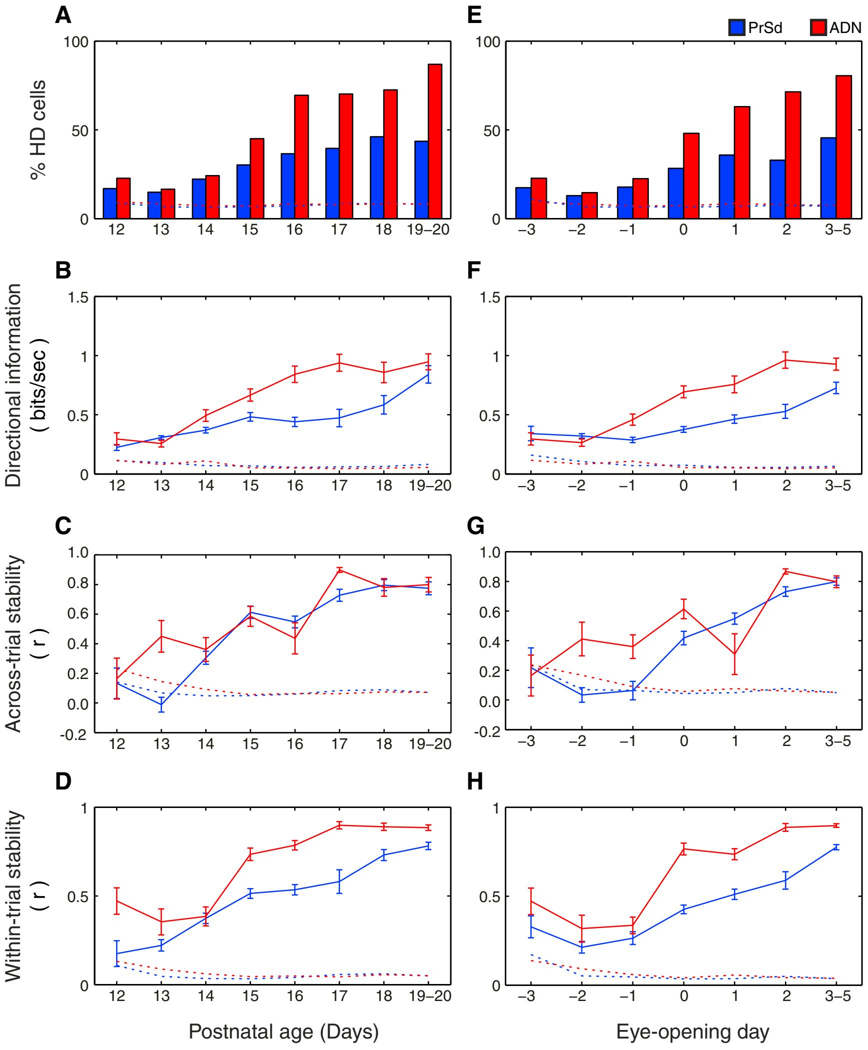

Figure 2. HD Responses Are Present from P12/E-3 and Mature Rapidly after Eye Opening

(A and E) Proportion of HD cells recorded from the PrSd (blue) and ADN (red), in rat pups aged between 12 and 20 days, expressed as a percentage of total recorded cells in each area, grouped by postnatal age (A) or eye-opening day (E). E0 marks the day of eye opening. Dashed lines represent the p = 0.05 significance level for the percentage of HD cells matched for brain area and age or eye-opening day (see Experimental Procedures).

(B–D and F–H) Spatial firing properties of HD cells recorded from the PrSd (blue) and ADN (red) as a function of age (B–D) and eye-opening day (F–H). Shown are DI per spike, expressed in bits/sec (B and F); across-trial stability between two consecutive trials (C and G); and within-trial stability between the first and second halves of one trial (D and H). Both stability measures are calculated as the spatial correlations (r) of the respective directional plots. Solid lines represent the mean over cells (±SEM). Dashed lines represent the p = 0.05 level of expected mean value of spatiality based on spike-shuffled data.

P12–P13 [10]. Auditory cues are therefore unlikely to support HD cell stability in very young animals. By contrast, olfaction is available to rats very early during development [19] and can exert control over adult HD cells [13], while tactile information could be gathered through active whisking, which emerges at around P10–P13 in the rat [20].

The HD circuit is widely modeled using a neural architecture called a continuous attractor network [21–25]. Where the development of spatial continuous attractors has been studied, models have relied on fixed, spatially tuned inputs to set up attractor connectivity as the network matures [26–28]. In the case of HD cells, this fixed reference input has been assumed to be a distal visual landmark [26, 27], as such landmarks remain in a fixed allocentric direction as the animal moves around the environment. Our results suggest, instead, that modalities other than vision may underpin the organization of the incipient HD circuit and that local rather than distal cues may play a critical role.

The Onset of Patterned Vision Prompts the Rapid Maturation of the HD System

Eye opening marks the onset of patterned vision, which affords access to discrete visual landmarks and optic flow. Coincident with eye opening, both the percentage of HD responses and their quality increase dramatically (Figures 2E–2H), suggesting that the sudden access to patterned visual information (both local and distal) spurs a sharp improvement in the HD system. The increase in HD cells at eye opening is particularly pronounced in the ADN, raising the possibility that visual input is critical to ADN HD responses.

A prominent distal landmark (only accessible via vision) can gain control over HD cell responses within 24 hr of eye opening. Given the protracted development of the visual system in the rat (with responses to visual stimuli in V1 still immature at P23 [29]), this is a striking demonstration of how rapidly visual information is incorporated into the HD system. Notably, the PFDs of simultaneously recorded cells appear to be coupled (Figures 3B and 3C) such that the angular difference between them stays constant [13] even upon rotation, supporting the view that a continuous attractor network architecture [21–25] may already be in place by the time of eye opening in the rat.

In summary, our findings at once confirm the significance of vision for accurate spatial representation, while challenging the prediction that it is required for the early organization of incipient spatial networks. We establish the possibility that an integrative sensory-motor circuit can develop in the absence of a sensory modality that is understood to be critical in its adult state yet is nonetheless primed to incorporate input from that modality immediately upon its availability.

Experimental Procedures

Subjects

37 male Lister Hooded rats (PrS n = 27; ADN n = 10), aged P10–P20 and weighing 18–29 g at the time of surgery, were used as subjects. Pups were checked at the beginning and end of each day for evidence of eye opening. The first day on which at least one of the eyelids had opened was labeled E0.

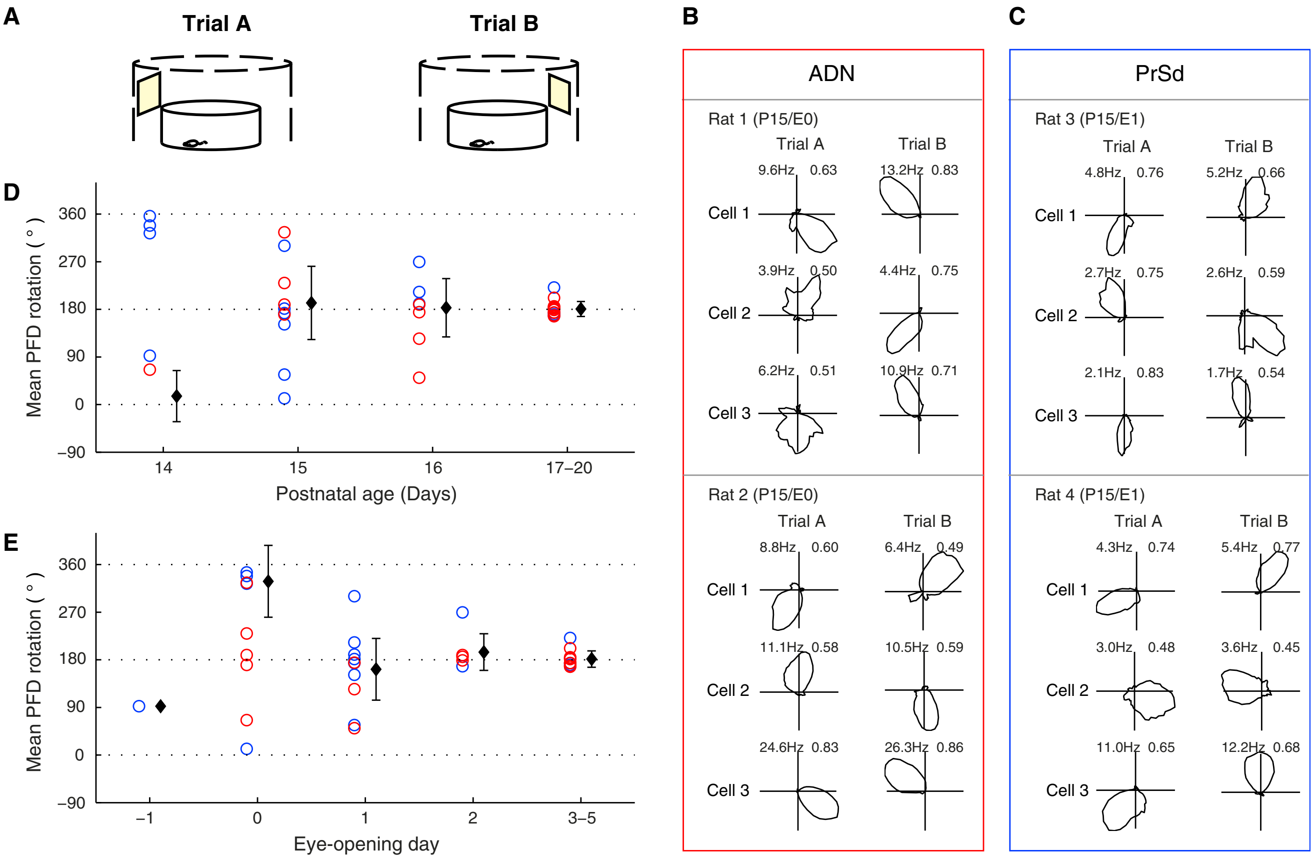

Figure 3. HD Cells Follow the Rotation of a Visual Landmark from as Early as P15/E0

(A) Schematic showing experimental method. A prominent visual landmark was rotated from its original position (Trial A) by 180°, to the opposite side of the recording environment (Trial B) (see Experimental Procedures for details).

(B and C) Earliest examples of simultaneously recorded HD cell ensembles over which a visual landmark exerts control (ADN, B; PrSd, C). Three example cells per ensemble are shown, recorded from the ADN (Rat 1, Rat 2) and the PrSd (Rat 3, Rat 4). Numbers at top left of polar plots indicate peak firing rate (Hz), and those at top right indicate RV (see Experimental Procedures).

(D and E) Mean rotations of preferred firing directions (PFDs), for all recorded ensembles of HD cells. Each circle represents the mean PFD rotation of an ensemble of simultaneously recorded HD cells; blue represents PrSd, and red represents ADN. In black, the grand mean (±SEM) of the rotations of all ensembles from each age (P) or eye-opening (E) group is shown.

Surgery and Electrodes

Rats were implanted with 4–8 tetrodes at the following stereotaxic coordinates: for ADN, 1.7 mm posterior to bregma, 1.2 mm lateral to the midline, and 4.2 mm ventral from bregma; for PrSd, 1.6 mm anterior to the sinus, 2.45 mm lateral to the midline, and 2.15 mm ventral from the cortical surface. Tetrode position was confirmed by postmortem Nissl staining.

Single-Unit Recording

Single-unit data were acquired using the DACQ system (Axona). Position and directional heading were recorded using a two-light tracking system, placed in a fixed orientation relative to the animal's head. Isolation of single units from tetrode-recorded data was performed manually on the basis of peak-to-trough amplitude and principal components using the TINT software package (Axona) with the aid of KlustaKwik [30] automated clustering.

Behavioral Testing

Single-unit recording trials took place in one of two recording arenas. (1) To test for the presence of HD cells and assess spatial firing properties, we used a square box (62.5 cm side length, 50 cm high), painted light gray and placed on a black platform within the open laboratory. (2) For visual landmark rotation trials, was used a light gray wooden cylinder (79 cm diameter, 50 cm high), centered within a circular set of black curtains. The only spatially polarizing cue was a white card, placed 55 cm outside the walls of the recording arena. On rotation trials, the white card was moved by 180°, while the floor and arena remained in the same position. Animals were disoriented before entering the curtained enclosure.

Classification of Single Units as HD Cells

Only sessions in which the linear path length exceeded 15.7 m, the angular path length exceeded the equivalent of 43 head turns, and cells fired at least 100 spikes in a recording session were included in further analysis. Single units were classified as HD cells if the mean resultant vector length (Rayleigh vector, RV) of the polar plot exceeded a threshold defined as the 95th percentile of a population of RV scores derived from age- and brain area-matched spatially shuffled data [5].

Quantitative Analysis of Directional Signaling

DI is an estimate of the mutual information I(R|X) between firing rate R and direction X. I(R|X) is divided by the overall mean firing rate of the cell in the trial, giving a final estimate in bits/spike [31]. Across-trial stability was defined as the correlation (Pearson's r) between spatially corresponding bins from two consecutive trials. Intra-trial stability was defined as the correlation between spatially corresponding bins from the first and second half of a single trial. The rotation of HD cell PFDs (following visual landmark rotation) was defined as the rotation of the baseline polar plot relative to the landmark rotation polar plot, which yielded the highest correlation between them.

Statistical Analysis

p = 0.05 levels for the percentages of HD cells were derived from a binomial distribution, based on the number of units recorded and assuming a false-positive HD classification rate of 5%. For all measures of directional signaling, the p = 0.05 levels were derived from the 95th percentiles of

distributions of mean directionality scores, drawn from matched numbers of spatially shuffled polar plots [5]. Developmental trends in HD cell firing were analyzed using a two-way ANOVA (age and area, or eye-opening day and area), and post hoc tests were SMEs. PFD rotation was assessed using the Watson-Williams test and the V test [32], with a specified response of 180°.

A detailed description of experimental methods can be found in Supplemental Experimental Procedures.

Supplemental Information

Supplemental Information includes Supplemental Experimental Procedures and two figures and can be found with this article online at http://dx.doi.org/10.1016/j.cub.2014.12.030.

Author Contributions

H.M.T., J.P.B., F.C., and T.J.W. designed the experiments and analyses. H.M.T. and J.P.B. collected the data, and H.M.T. and T.J.W. analyzed the data. H.M.T. assembled the figures, and all authors contributed to drafting the manuscript.

Acknowledgments

We acknowledge funding from the ERC (DEVSPACE grant to F.C.), the BBSRC (grant BB/I021221/1 to F.C.), the Royal Society (URF fellowship to T.J.W.), the Agency for Science, Technology and Research (Singapore) (PhD studentship to H.M.T.), the Gatsby Charitable Foundation, and the Wellcome Trust (to J.O.). We thank Kate Jeffery for use of laboratory space in the Institute of Behavioural Neuroscience, UCL.

Received: November 21, 2014
Revised: December 5, 2014
Accepted: December 9, 2014
Published: February 5, 2015

References

1. Taube, J.S., Muller, R.U., and Ranck, J.B., Jr. (1990). Head-direction cells recorded from the postsubiculum in freely moving rats. I. Description and quantitative analysis. J. Neurosci. *10*, 420–435.
2. Taube, J.S. (1995). Head direction cells recorded in the anterior thalamic nuclei of freely moving rats. J. Neurosci. *15*, 70–86.
3. Taube, J.S. (2007). The head direction signal: origins and sensory-motor integration. Annu. Rev. Neurosci. *30*, 181–207.
4. Taube, J.S., Muller, R.U., and Ranck, J.B., Jr. (1990). Head-direction cells recorded from the postsubiculum in freely moving rats. II. Effects of environmental manipulations. J. Neurosci. *10*, 436–447.
5. Wills, T.J., Cacucci, F., Burgess, N., and O'Keefe, J. (2010). Development of the hippocampal cognitive map in preweanling rats. Science *328*, 1573–1576.
6. Langston, R.F., Ainge, J.A., Couey, J.J., Canto, C.B., Bjerknes, T.L., Witter, M.P., Moser, E.I., and Moser, M.B. (2010). Development of the spatial representation system in the rat. Science *328*, 1576–1580.
7. Altman, J., and Sudarshan, K. (1975). Postnatal development of locomotion in the laboratory rat. Anim. Behav. *23*, 896–920.
8. Renner, M.J., and Pierre, P.J. (1998). Development of exploration and investigation in the Norway rat (Rattus norvegicus). J. Gen. Psychol. *125*, 270–291.
9. Bjerknes, T.L., Moser, E.I., and Moser, M.-B. (2014). Representation of geometric borders in the developing rat. Neuron *82*, 71–78.
10. Alberts, J.R. (1984). Sensory-perceptual studies in the Norway rat: a view toward comparative studies. In Comparative Perspectives on Memory Development, R. Kail and N.S. Spear, eds. (Erlbaum), pp. 65–102.
11. Stackman, R.W., and Taube, J.S. (1997). Firing properties of head direction cells in the rat anterior thalamic nucleus: dependence on vestibular input. J. Neurosci. *17*, 4349–4358.
12. Stackman, R.W., Clark, A.S., and Taube, J.S. (2002). Hippocampal spatial representations require vestibular input. Hippocampus *12*, 291–303.
13. Goodridge, J.P., Dudchenko, P.A., Worboys, K.A., Golob, E.J., and Taube, J.S. (1998). Cue control and head direction cells. Behav. Neurosci. *112*, 749–761.
14. Zugaro, M.B., Arleo, A., Berthoz, A., and Wiener, S.I. (2003). Rapid spatial reorientation and head direction cells. J. Neurosci. *23*, 3478–3482.
15. Goodridge, J.P., and Taube, J.S. (1995). Preferential use of the landmark navigational system by head direction cells in rats. Behav. Neurosci. *109*, 49–61.
16. Blair, H.T., and Sharp, P.E. (1996). Visual and vestibular influences on head-direction cells in the anterior thalamus of the rat. Behav. Neurosci. *110*, 643–660.
17. Zugaro, M.B., Tabuchi, E., and Wiener, S.I. (2000). Influence of conflicting visual, inertial and substratal cues on head direction cell activity. Exp. Brain Res. *133*, 198–208.
18. Arleo, A., Déjean, C., Allegraud, P., Khamassi, M., Zugaro, M.B., and Wiener, S.I. (2013). Optic flow stimuli update anterodorsal thalamus head direction neuronal activity in rats. J. Neurosci. *33*, 16790–16795.
19. Polan, H.J., and Hofer, M.A. (1998). Olfactory preference for mother over home nest shavings by newborn rats. Dev. Psychobiol. *33*, 5–20.
20. Landers, M., and Philip Zeigler, H. (2006). Development of rodent whisking: trigeminal input and central pattern generation. Somatosens. Mot. Res. *23*, 1–10.
21. Skaggs, W.E., Knierim, J.J., Kudrimoti, H., and McNaughton, B.L. (1995). A model of the neural basis of the rat's sense of direction. In Neural Information Processing Systems 7, S.J. Hanson, J.D. Cowan, and C.L. Giles, eds. (MIT Press), pp. 173–180.
22. Redish, A.D., Elga, A.N., and Touretzky, D.S. (1996). A coupled attractor model of the rodent head direction system. Network *7*, 671–685.
23. Zhang, K. (1996). Representation of spatial orientation by the intrinsic dynamics of the head-direction cell ensemble: a theory. J. Neurosci. *16*, 2112–2126.
24. Goodridge, J.P., and Touretzky, D.S. (2000). Modeling attractor deformation in the rodent head-direction system. J. Neurophysiol. *83*, 3402–3410.
25. Song, P., and Wang, X.-J. (2005). Angular path integration by moving "hill of activity": a spiking neuron model without recurrent excitation of the head-direction system. J. Neurosci. *25*, 1002–1014.
26. Stringer, S.M., Trappenberg, T.P., Rolls, E.T., and de Araujo, I.E.T. (2002). Self-organizing continuous attractor networks and path integration: one-dimensional models of head direction cells. Network *13*, 217–242.
27. Hahnloser, R.H. (2003). Emergence of neural integration in the head-direction system by visual supervision. Neuroscience *120*, 877–891.
28. Widloski, J., and Fiete, I.R. (2014). A model of grid cell development through spatial exploration and spike time-dependent plasticity. Neuron *83*, 481–495.
29. Fagiolini, M., Pizzorusso, T., Berardi, N., Domenici, L., and Maffei, L. (1994). Functional postnatal development of the rat primary visual cortex and the role of visual experience: dark rearing and monocular deprivation. Vision Res. *34*, 709–720.
30. Harris, K.D., Henze, D.A., Csicsvari, J., Hirase, H., and Buzsáki, G. (2000). Accuracy of tetrode spike separation as determined by simultaneous intracellular and extracellular measurements. J. Neurophysiol. *84*, 401–414.
31. Skaggs, W.E., McNaughton, B.L., Gothard, K.M., and Markus, E.J. (1993). An information-theoretic approach to deciphering the hippocampal code. Adv. Neural Inf. Process. Syst. *5*, 1030–1037.
32. Zar, J.H. (2010). Biostatistical Analysis, Fifth Edition (Prentice Hall).

Current Biology
Supplemental Information

The Development of the Head Direction System before Eye Opening in the Rat

Hui Min Tan, Joshua Pope Bassett, John O'Keefe, Francesca Cacucci, and Thomas Joseph Wills

A

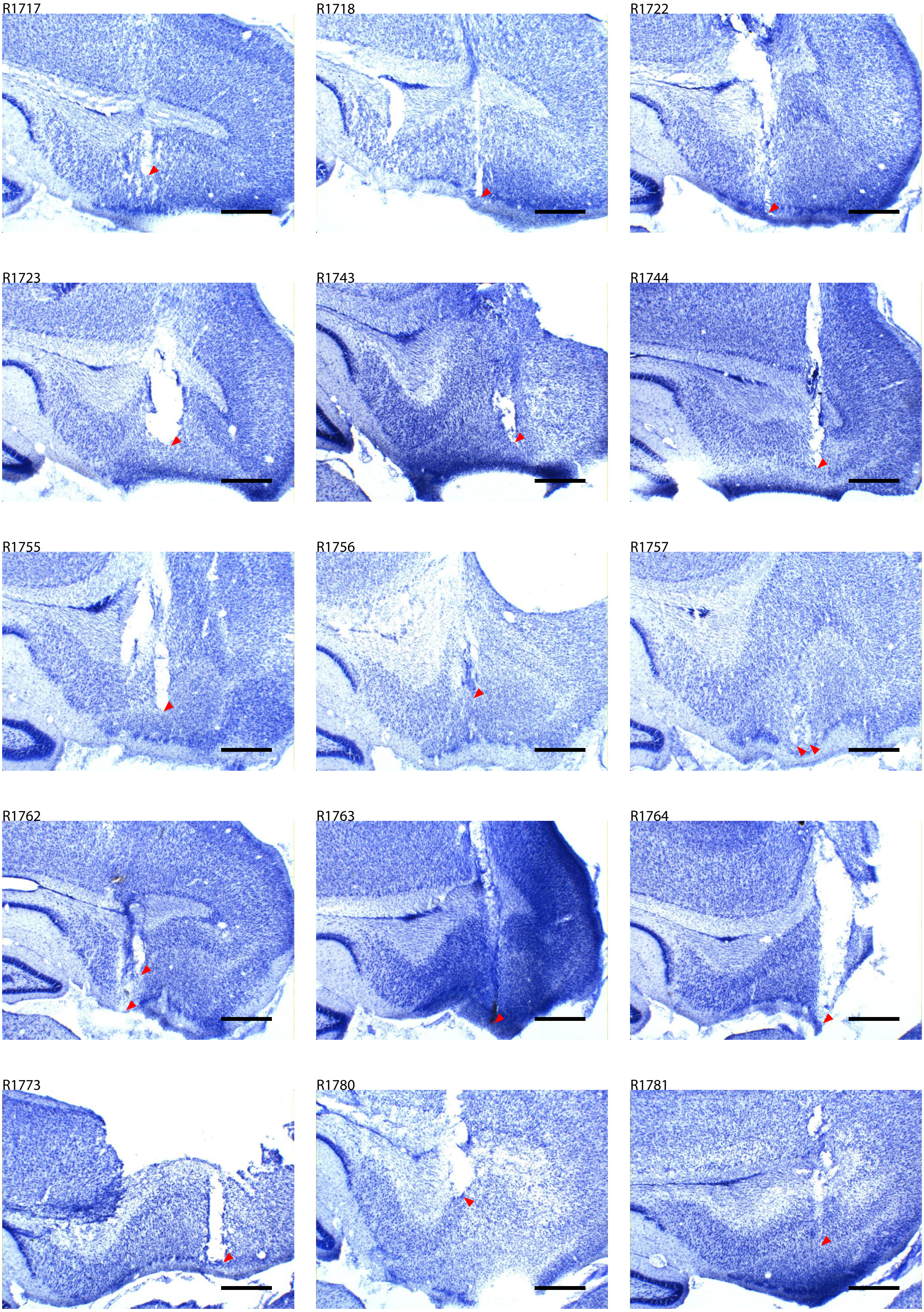
R1717
R1718
R1722
R1723
R1743
R1744
R1755
R1756
R1757
R1762
R1763
R1764
R1773
R1780
R1781

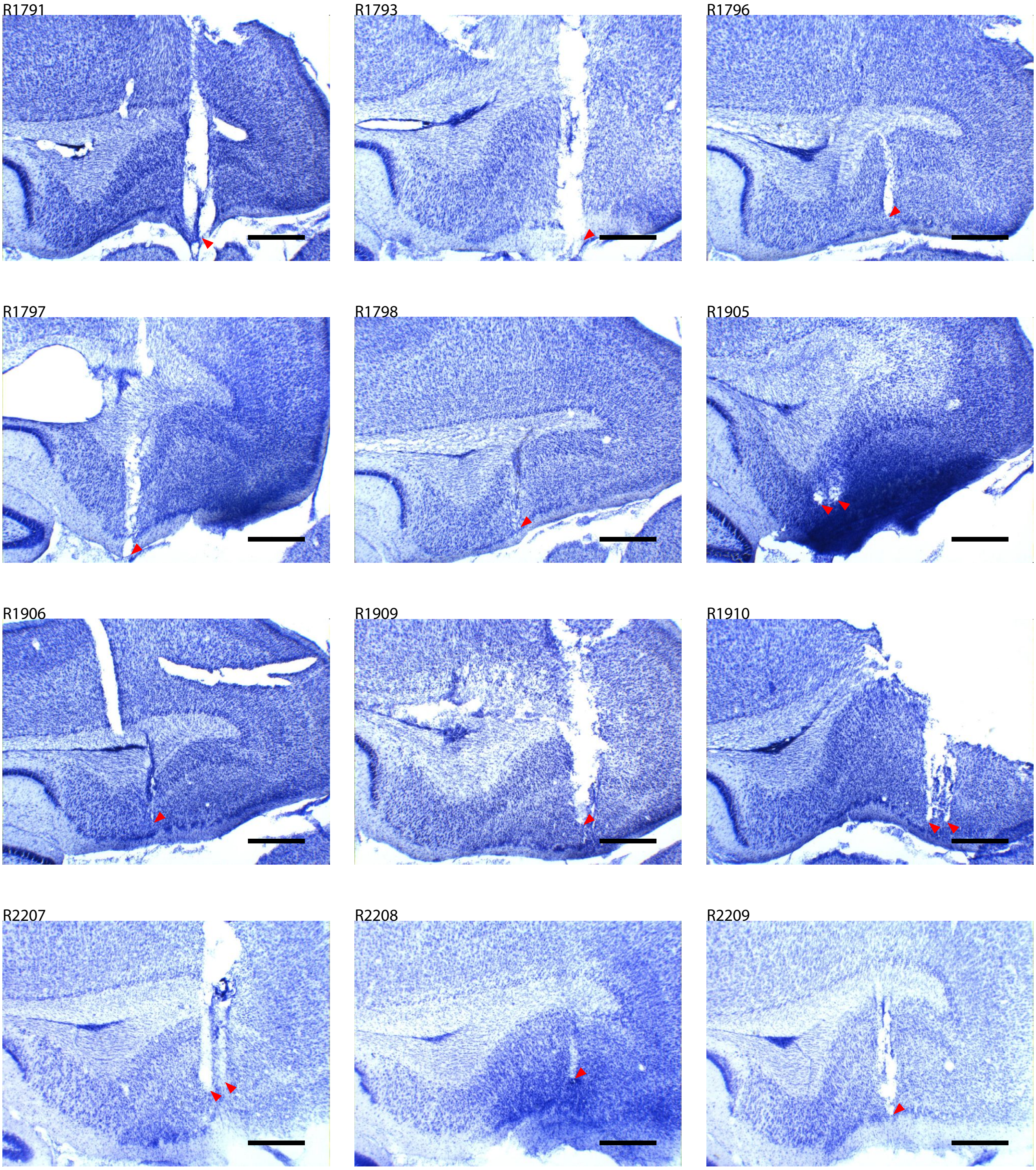
R1791
R1793
R1796
R1797
R1798
R1905
R1906
R1909
R1910
R2207
R2208
R2209

B

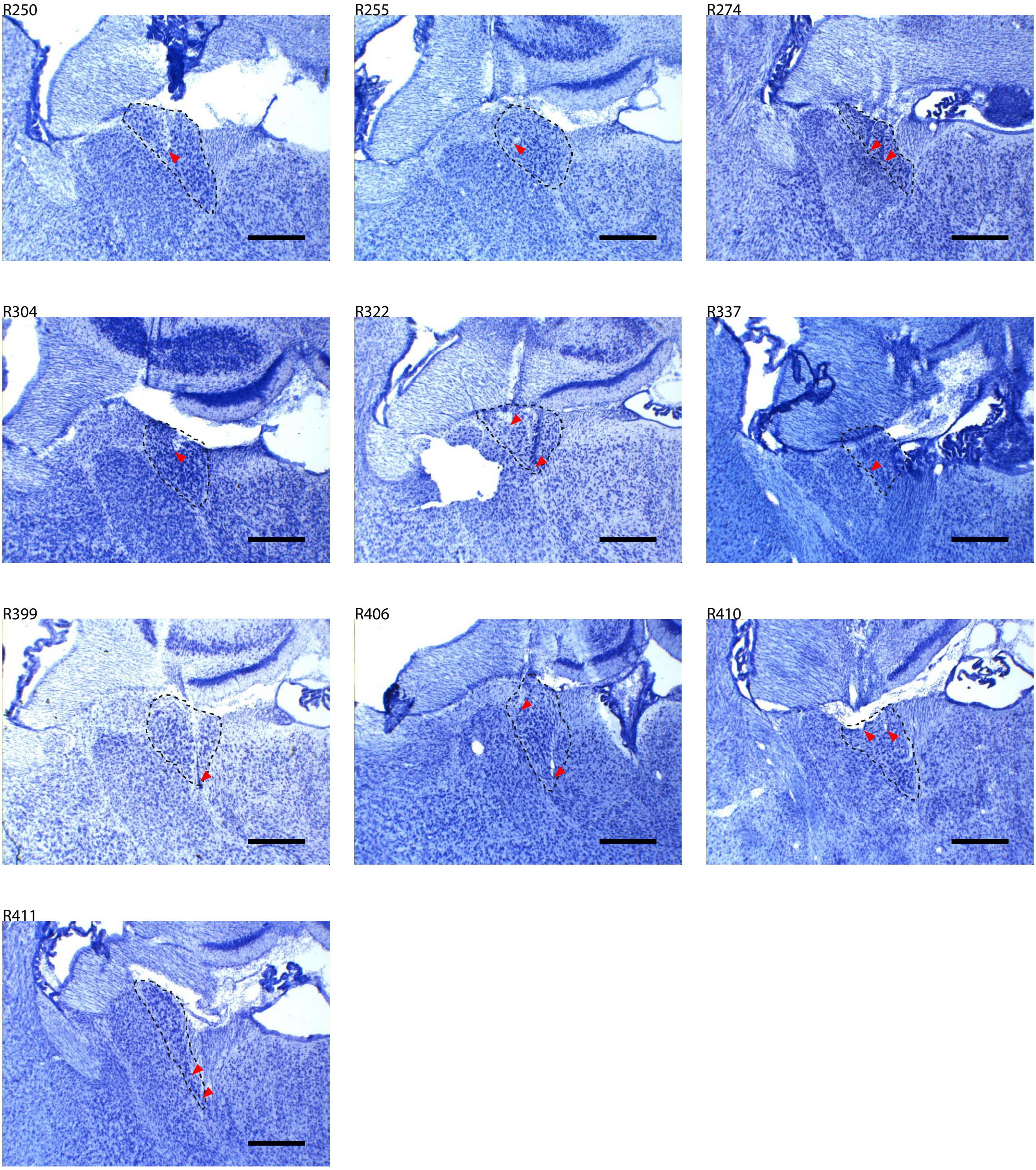

Figure S1, related to Figure 1. Nissl-stained brain sections showing representative recording locations in the PrSd (sagittal sections, n=27, panel A) and ADN (coronal sections, n=10, panel B). Red arrows mark the deepest location through which any given tetrode passed within the PrSd or ADN (the limits of the ADN are traced by dashed lines). Scale bars represent 500um.

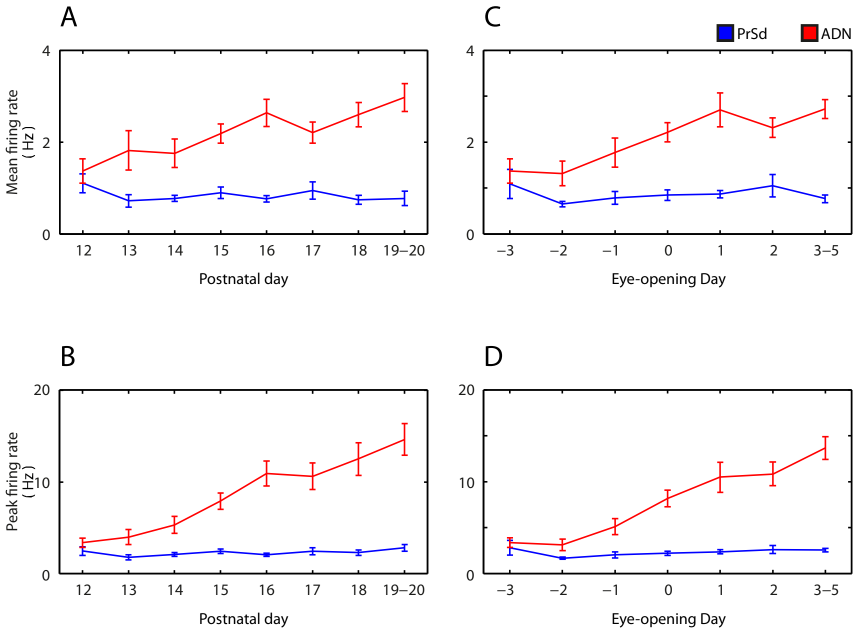

Figure S2, related to Figure 2. Firing rates of ADN HD cells increase during development to higher levels than those of PrSd HD cells. (A,C) Mean firing rates (mean ±SEM) of HD cells recorded from PrSd (blue) and ADN (red), grouped by postnatal day (Panel A) or eye-opening day (Panel C). (B,D) Peak firing rates (mean ±SEM) of HD cell polar plots from PrSd and ADN, grouped by postnatal day (Panel B) or eye-opening day (Panel D).

Supplemental Experimental Procedures

Subjects 37 male Lister Hooded rats (PrS n = 27; ADN n = 10), aged P10-P20, weighing 18-29g at the time of surgery, were used as subjects. Litters were bred on-site and implanted subjects remained with their mothers and litter-mates throughout the experimental period. Litters were housed in 42x32x21cm cages furnished with nesting material and environmental enrichment objects, and maintained on a 12:12 hour light:dark schedule with lights off at 13:00. Litters were culled to 8 pups at P4 in order to minimise inter-litter variability. Implanted pups were separated from the dam and littermates for around 20 to 120 minutes per day for electrophysiological recordings. Pups were checked at the beginning and end of each day for evidence of eye-opening. The first day on which at least one of the eyelids had opened was labelled E0.

Surgery and electrodes Rats were anaesthetised using 1-3% isoflurane and buprenorphine via subcutaneous injection at 0.15mg/kg of body weight. Rats were implanted with 4-8 tetrodes consisting of HM-L coated 90% platinum/10% iridium 17μm wire (California Fine Wire, Grover City, CA). The implanted apparatus weighed 1 gram. Tetrode bundles were implanted at the following stereotaxic coordinates, for ADN: 1.7 mm posterior to bregma, 1.2mm lateral from the midline at bregma, and 4.2mm ventral from bregma. For PrSd: 1.6mm anterior to the sinus; 2.45 mm lateral from the midline at the sinus and 2.15mm ventral to the cortical surface. Following surgery, rats were placed on a heating pad until they could move spontaneously and were then returned to the home cage. After experiments were completed, tetrode positions were confirmed by transcardially perfusing the rat (4% Paraformaldehyde in PBS) whilst the tetrodes remained in their final position, followed by brain sectioning at 30um, and Nissl-staining of the resulting sections.

Single unit recording Following surgery, rats were allowed 24 hours recovery. Tetrode bundles were then advanced ventrally in increments of 62.5-250 μm/day. Experimental recording sessions began when any single unit neural activity could be identified. Single unit data were acquired using the DACQ system (Axona Ltd, St. Albans, UK). Position and directional heading were recorded using a 2-point tracking system consisting of 2 LEDs spaced 7 cm apart and attached to the headstage amplifier in a fixed orientation relative to the animals' head. Isolation of single units from tetrode-recorded data was performed manually on the basis of peak-to-trough amplitude, or principal components using the TINT software package (Axona Ltd., St Albans, UK) with the aid of KlustaKwik [1] automated clustering. When two or more recording sessions were performed in the same day, isolated single units were treated as distinct cells if a) electrodes had been moved at least 125μm between sessions, b) at least 2 hours had elapsed between sessions, and c) the distributions of spike clusters in

waveform amplitude plots were clearly unrelated between the two sessions. Overnight, even if electrodes had not been moved, distributions of spike clusters were rarely related between the two days. Single units recorded across days were treated as independent cells unless there was similarity in either a) distributions of spike clusters on a tetrode or b) the spatial firing correlates of the cells. If single units were found to be repeatedly sampled over sessions, only data from the first session were included in the analyses. However, due to the inherent limitations of the tetrode recording technique, it should be noted that a small number of the single units in this study may represent re-sampled neurons, notwithstanding the above precautions.

Behavioural Testing Single-unit recording trials took place in one of two recording arenas. (1) To test for the presence of HD cells, and assess their spatial firing properties, recordings were performed in a square box (62.5cm side length, 50cm high) painted light grey, placed on a black platform. The box was placed in the open laboratory, and distal visual cues were available in the form of the fittings and contents of the laboratory. The floor of the arena was not cleaned. There were no further polarising cues placed within the recording arena. Rats were subject to between 1 and 4 standard recording trials per session. (2) To assess whether HD cells followed a visual landmark rotation, recordings were performed in a light-grey wooden cylinder (79cm diameter, 50cm high) placed on a black platform, centred within a set of black curtains hanging from a circular track on the ceiling. A single, spatially-polarising cue in the form of a white card measuring 102 x 77 cm was hung within the curtains, but 55 cm distal to and visible above the walls of the recording arena, with a 40 watt lamp directed at the card. Every time rat pups entered the curtained enclosure, they were purposely disoriented by gently turning them in a closed opaque box and released in the cylindrical recording box always facing in the same direction with respect to the laboratory frame of reference. Rat pups were kept in a separate holding box (40 x 40 x 5cm) furnished with bedding and a heating pad in between recording trials. For landmark rotation trials, the white cue card and lamp were moved to the opposite side of the recording arena, whilst the rat remained outside of the curtains. The floor and walls of the recording arena were not rotated when the landmark was rotated, though the floor was cleaned.

Construction of polar tuning curves To minimise artefactual correlates due to under-sampling of position, data were included in further analyses only if the linear path length for the session exceeded 15.7m, and the angular path length exceeded the equivalent of 43 full head turns (values derived from the 10th percentile of the whole dataset). Directional data were sorted into 6° bins in the yaw plane. Following this, total dwell time, d, and spike count, s, for the whole trial was calculated for each directional bin. The binned dwell time and spike counts were then smoothed using a 30° boxcar filter, and the rate for each directional bin is defined as s/d.

Classification of single-units as HD cells To minimise artefactual correlates due to under-sampling, only cells which fired at least 100 spikes in a recording session were included in further analyses. The mean resultant vector length (Rayleigh vector; RV) was calculated for the polar plot of each cell as follows: The firing rates in each bin of the polar plot were treated as grouped directional data ([2], p613). The rectangular co-ordinates X and Y of the mean (resultant) vector of the polar plot were calculated as:

$$X = \frac{\sum f_i cos\ a_i}{n}$$

$$Y = \frac{\sum f_i sin\ a_i}{n}$$

where f_i is the firing rate in each polar plot bin, a_i the angle of that bin and n the total summed rate for the polar plot. The length of the resultant vector, R, was then defined as:

$$R = \sqrt{X^2 + Y^2}$$

R is limited between 0 (no angular bias) and 1 (all firing concentrated in one directional bin), and is not affected by the overall firing rate of the cell. Single units were classified as HD cells if the RV of the polar tuning curve exceeded a threshold defined as the 95th percentile of a population of RV scores derived from age- and brain area-matched spatially shuffled data [3]. Briefly, shuffled data were generated by shifting spike trains relative to position by a random amount between 20 seconds and trial duration minus 20 seconds, leaving the temporal structure of the spike train and the positional data otherwise unchanged. The shuffled data were then used to construct polar plots, as described above. This process was repeated a sufficient number of times for there to be 100,000 shuffled RV values for every 1-day age or eye-opening group, for each brain area. Single units with an RV ≥ 95th percentile of this shuffled population were defined as HD cells.

Quantitative analysis of directional signalling *Directional information* is a measure of the extent to which a cell's firing can be used to predict the direction of the animal's head in bits/spike. The estimate of the mutual information I(R|X) between firing rate R and direction X is:

$$I(R|X) \approx \sum_i p(\vec{x_i}) f(\vec{x_i}) \log_2 \left(\frac{f(\vec{x_i})}{F} \right)$$

where $p(\vec{x_i})$ is the probability for the animal facing direction $\vec{x_i}$, $f(\vec{x_i})$ is the firing rate observed at $\vec{x_i}$, and F is the overall firing rate of the cell. I(R|X) is then divided by the overall mean firing rate of the cell in the trial,

giving an estimate in bits/spike [4]. *Across-trial stability* was defined as the correlation (Pearson's r) between spatially corresponding bins from two consecutive trials, using only those bins in which firing rate >0 Hz in at least one trial. Trial pairs were used for assessing across-trial stability if a single unit was classified as a HD cell on the first trial of the pair. *Intra-trial stability* was defined as the correlation between spatially corresponding bins from the first and second half of a single trial, using only those bins in which firing rate > 0Hz in at least one half of the trial. To define the rotation of HD cell preferred direction (following visual landmark rotation, Figure 3) the polar plot for the rotated landmark condition was correlated against that for the baseline condition, whilst rotating the baseline polar plot in 6° steps. The rotation of the baseline polar plot that produced the highest r-value was taken to define the rotation of the preferred direction of the cell. Single units were used for the preferred direction rotation analysis if they were defined as HD cells in (a) the 'baseline' visual landmark condition AND (b) either or both of two 'standard' trials (outside the curtained environment) that preceded the 'baseline' visual landmark trial.

Calculation of p = 0.05 levels for the percentage of HD cells, and analysis of directional signalling P=0.05 levels for the percentage of single units classified as HD cells (dashed lines on Figure 2A, E) were generated as follows: if M polar plots per rat were analysed for a given age and brain area, then the mean percentage of HD cells expected to be found at most once per 20 experiments under the null hypothesis (non-directional firing) was defined as the 95th percentile of a binomial distribution based on M samples and a 5% success probability. The P=0.05 levels for the measures of directional signalling for each age and brain area (dashed lines, Figure 2B, C, D, F, G, H) were derived as follows: if M polar plots contributed to the mean value for a given age and area, M polar plots were sub-sampled at random from the shuffled population (see above) for that age/region and the mean value of directional signalling found. This was repeated 200,000 times, generating a distribution of the mean scores expected from populations (of size M) of randomised data. The P = 0.05 level of spatial firing was defined as the 95th percentile of this population.

Statistical Analysis Developmental trends in the spatial characteristics of HD cell firing (Figure 2B, C, D, F, G, H) were initially analysed using a 2-way ANOVA (Postnatal age, Area) or (Eye-opening day, Area) for each measure of spatial tuning or stability. Post-hoc tests were conducted using Simple Main Effects. The developmental trend for HD cell ensembles to follow a landmark rotation were assessed using the Watson-Williams test (a circular analog of ANOVA). To test whether HD cell ensembles significantly followed landmark rotation on individual days, we used the V-test [2], a variation of the Rayleigh test for non-uniformity, in which a hypothesised response direction is specified, in this case 180°. To test for changes in inter-animal

variance of mean PFD rotations between days, we used the Watson's two-sample test for homogeneity, which tests whether two circular datasets have been drawn from populations that share the same distribution [5].

Supplemental References

1. Harris, K. D., Henze, D. A., Csicsvari, J., Hirase, H., and Buzsaki, G. (2000). Accuracy of tetrode spike separation as determined by simultaneous intracellular and extracellular measurements. J.Neurophysiol. *84*, 401–414.

2. Zar, J. H. (2010). Biostatistical Analysis 5th ed. (Prentice Hall).

3. Wills, T. J., Cacucci, F., Burgess, N., and O'Keefe, J. (2010). Development of the hippocampal cognitive map in preweanling rats. Science (80-.). *328*, 1573–1576.

4. Skaggs, W. E., McNaughton, B. L., Gothard, K. M., and Markus, E. J. (1993). An information-theoretic approach to deciphering the hippocampal code. Adv Neural Inf Process Syst *5*, 1030–1037.

5. Jammalamadaka, S. R., and Sengupta, A. (2001). Topics in Circular Statistics (World Scientific).
